# The Potential Pathogenicity of Myelin Oligodendrocyte Glycoprotein Antibodies in the Optic Pathway

**DOI:** 10.1097/WNO.0000000000001772

**Published:** 2022-12-08

**Authors:** Magdalena Lerch, Angelika Bauer, Markus Reindl

**Affiliations:** Clinical Department of Neurology, Medical University of Innsbruck, Innsbruck, Austria.

## Abstract

Myelin oligodendrocyte glycoprotein (MOG) antibody-associated disease (MOGAD) is an acquired inflammatory demyelinating disease with optic neuritis (ON) as the most frequent clinical symptom. The hallmark of the disease is the presence of autoantibodies against MOG (MOG-IgG) in the serum of patients. Whereas the role of MOG in the experimental autoimmune encephalomyelitis animal model is well-established, the pathogenesis of the human disease and the role of human MOG-IgG is still not fully clear.

**Evidence Acquisition:**

PubMed was searched for the terms “MOGAD,” “optic neuritis,” “MOG antibodies,” and “experimental autoimmune encephalomyelitis” alone or in combination, to find articles of interest for this review. Only articles written in English language were included and reference lists were searched for further relevant papers.

**Results:**

B and T cells play a role in the pathogenesis of human MOGAD. The distribution of lesions and their development toward the optic pathway is influenced by the genetic background in animal models. Moreover, MOGAD-associated ON is frequently bilateral and often relapsing with generally favorable visual outcome. Activated T-cell subsets create an inflammatory environment and B cells are necessary to produce autoantibodies directed against the MOG protein. Here, pathologic mechanisms of MOG-IgG are discussed, and histopathologic findings are presented.

**Conclusions:**

MOGAD patients often present with ON and harbor antibodies against MOG. Furthermore, pathogenesis is most likely a synergy between encephalitogenic T and antibody producing B cells. However, to which extent MOG-IgG are pathogenic and the exact pathologic mechanism is still not well understood.

Myelin oligodendrocyte glycoprotein (MOG), a minor component of myelin in the central nervous system (CNS), is expressed in the outermost layer of myelin (1). It is a Type 1 integral membrane glycoprotein of 26–28 kDa, only found in mammals and is highly conserved between species (2,3). Up to 15 splice variants have been described in humans and nonhuman primates, but not in rodents, that mainly differ in their cytoplasmatic domains (4). Despite intensive research, the function of MOG still remains to be fully determined. Postulated biological roles include an adhesion molecule, a compactor of myelin, or a stabilizer of microtubules (5,6). Furthermore, it has been shown to interact with C1q, nerve growth factor, dendritic-cell (DC)-specific intercellular adhesion molecule-3 grabbing nonintegrin, and to be a cellular receptor for rubella virus (7,8,9,10). The extracellular site is composed of an immunoglobulin (Ig)-V-like domain that is highly immunogenic and can evoke inflammatory demyelinating immune responses. It was used extensively to induce inflammation in experimental autoimmune encephalomyelitis (EAE), a proposed animal model of multiple sclerosis (MS). However, the use of cell-based assays with full-length natively-folded MOG for the detection of human MOG immunoglobulin G antibodies (MOG-IgG) in patients with acquired demyelinating diseases (ADS) showed that MOG-IgG-associated disease (MOGAD) represents a disease distinct from MS (1,11).

MOGAD is a rare disease with an incidence of 0.16/100,000 people (12), but the spectrum of clinical symptoms is ever expanding. The most common presentations are optic neuritis (ON), acute disseminated encephalomyelitis (ADEM), transverse myelitis, aquaporin 4 (AQP4)-IgG negative neuromyelitis optica spectrum disorders (NMOSD), brainstem syndrome, and cortical encephalitis (13). Moreover, there is a correlation between age and clinical presentation, with ADEM being more common in children and optico-spinal lesions being more present in adults (12,14,15,16,17,18,19,20,21,22).

Despite the increasing knowledge of clinical MOGAD presentations, the pathophysiology and importantly, the pathogenic role of human MOG-IgG, remains to be fully determined. This review aims to summarize present studies on MOG-IgG pathology and pathogenesis of this rare inflammatory demyelinating disease with a focus on optic pathway involvement.

## HUMAN MYELIN OLIGODENDROCYTE GLYCOPROTEIN-IgG—DETECTION AND BINDING TO MYELIN OLIGODENDROCYTE GLYCOPROTEIN

The introduction of state-of-the-art cell-based assays for the detection of human MOG-IgG resulted in the characterization of a novel subset of ADS different from MS and NMOSD (1,11,23). Importantly, only antibodies recognizing conformational epitopes present on the full-length protein were found to be of clinical interest (11,24). Therefore, the use of linear peptides or unfolded proteins in ELISA and immunoblots is not suitable for detection of MOG-IgG in human serum samples (11,25). The epitopes recognized most frequently in human MOG are located within the extracellular IgV-like domain and are heterogenic. Proline 42 is the most important amino acid for antibody recognition, located in the CC′ loop, followed by histidine 103 and serine 104 (26,27). The latter constitutes the main binding site of the monoclonal antibody 8-18-C5 (28). Most human MOG-IgGs are not or only weakly cross-reactive with rodent MOG, with the important P42S mutation, which hampers investigation in rodent models (26,29,30). Moreover, it has been shown that in patients with persisting MOG-IgG serostatus, the epitope remains constant (26).

Human MOG-IgG has a reduced binding to paraformaldehyde-treated MOG (27). This further supports the dependence on binding to natively-folded conformational epitopes. MOG has a glycosylation site at asparagine 31 and studies have shown conflicting results regarding MOG-IgG binding in the absence of the glycan. Using the mutant N31D, some serum samples revealed better recognition of MOG (23,26,31). Nevertheless, another study additionally using the mutant N31A found that 60% of MOG-IgG binding was altered (32). The human MOG gene undergoes alternative splicing and distinct MOG isoforms, that differ in their cytoplasmatic domain, have been described (33,34,35,36). Intriguingly, the hydrophobic cytoplasmatic membrane-associated domain was recently described to play a pivotal role for the recognition of human MOG-IgG and the authors propose that this domain generates a certain distance between distinct MOG proteins enabling bivalent binding of MOG-IgG (37). A recent study investigated the binding of MOG-IgG to 6 major MOG isoforms. A third of all patient samples only recognized MOGα_1_ and MOGβ_1_, both of which have this hydrophobic domain. However, most of the samples recognized all or most MOG isoforms tested, despite the lack of this domain (38). These findings reveal that human MOG-IgG has a complex and dynamic epitope specificity.

## T- AND B-CELL MEDIATED PATHOGENESIS OF MYELIN OLIGODENDROCYTE GLYCOPROTEIN-ASSOCIATED DISEASE

The encephalitogenic role of MOG has been analyzed since decades because it is frequently used as an autoantigen in the EAE model of CNS demyelination (39,40,41,42). In this model, animals are actively immunized with different myelin proteins/peptides or are used for passive transfer experiments to study the underlying immunopathogenesis. Dependence on T and B cells and their orchestration is highly mediated by the type of antigen (i.e., the specific myelin protein, recombinant protein/peptide) and the genetic background of animals. MOG-IgG has been shown to enhance T-cell mediated disease in some animal models, whereas B cells were demonstrated to be unimportant for disease development in other animals (reviewed in Refs. 1,^43^).

In patients with MOGAD, genetic studies showed no strong correlation between human leukocyte antigen (HLA) genotype and MOGAD development; importantly, no cause for disease pathogenesis has been found. A recent study found a protective effect of the HLA-C*03:04 allele (44), whereas a Dutch study could not find any associations (45). In addition, in a Chinese cohort, there was an association between pediatric-onset MOGAD for DQB1*05:02-DRB1*16:02, but not for adult MOGAD (46). Moreover, in some cases, a viral infection preceded MOGAD diagnosis: Epstein–Barr virus, herpes simplex virus 1, rubella, varicella zoster virus, and severe acute respiratory syndrome–coronavirus-2 (47,48,49,50,51,52). A rare paraneoplastic incidence of MOGAD has also been described (53). A few patients developed MOGAD while given tumor necrosis factor-α (TNFα) inhibitors, yet this is an uncommon phenomenon (54).

**FIG. 1.**
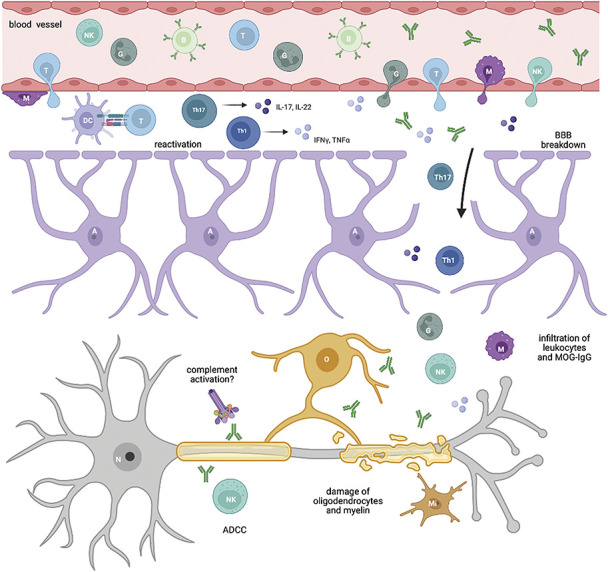
Pathogenesis of MOGAD. Encephalitogenic T cells gain access into the CNS (mostly through the meninges), where they seek contact with APC (DC) and get reactivated. After polarization toward Th1 and Th17 T-cell subsets, subsequent release of cytokines leads to a breakdown of the BBB and to a massive infiltration of other leukocytes and MOG-IgG, that were produced in the periphery. MOG-IgG bind to their target on the surface of oligodendrocytes and on myelin and can be directly pathogenic via ADCC, altering the cytoskeleton of oligodendrocytes or also complement activation. The synergy of activated immune cells and MOG-IgG eventually leads to damage of oligodendrocytes and to demyelination. A indicates astrocyte; ADCC, antibody-dependent cellular cytotoxicity; B, B-cell; BBB, blood–brain barrier; DC, dendritic cell; G, granulocyte; IFNγ, interferon-γ; IL, interleukin; M, macrophage; Mi, microglia; MOG, myelin oligodendrocyte glycoprotein; N, neuron; NK, natural killer cell; O, oligodendrocyte; T, T cell; Th1, T-helper 1 cell; Th17, T-helper 17 cell; TNFα, tumor necrosis factor-α. Created with BioRender.com.

Similar to the EAE animal, in human MOGAD, a synergy between encephalitogenic T cells and B cells is observed (Fig. 1). Under normal circumstances, the CNS parenchyma is free of lymphocytes. In recent years, however, it has become clear that neuroimmune interactions are important for CNS homeostasis (55,56,57). A pro-inflammatory environment that enables opening of the blood–brain barrier (BBB) for the entry of potentially pathogenic antibodies is crucial for the pathogenesis of MOGAD. There are 2 possible explanations for the generation of autoimmune responses against MOG: the “inside-out” hypothesis postulates primary damage of oligodendrocytes that leads to drainage of myelin antigens into lymph nodes (LN) via lymphatic vessels of the dura mater (58,59,60,61). These are transported as soluble antigens or by DC of the choroid plexus or the meninges into the deep cervical LN, where antigen presentation and priming of T cells takes place (60,62,63,64,65,66,67). However, drainage of foreign antigens via this pathway has also been shown to induce tolerance of immune cells toward self-antigens rather than autoimmune activation; therefore, it is possible that a certain threshold of drained CNS antigens has to be reached (68,69,70,71). The second possibility, called the “outside-in” hypothesis, posits an activation of lymphocytes in peripheral LN through molecular mimicry or pan-activation after a systemic viral infection (65). Cross-reactivity has been shown between MOG-IgG and butyrophilin, a milk protein (72). Furthermore, negative thymic selection of T cells toward MOG self-tolerance is believed to be incomplete (73,74). In line, a lack of immune tolerance toward MOG has been shown in knock-out mice (75) and immune tolerance was restored using mRNA-based vaccination with MOG peptides or transgenic expression of MOG within immune cells (76,77).

In both cases, T-cells home to the brain after priming where they most likely enter the brain parenchyma through the meninges or the choroid plexus (55,65,78,79). After entry into the CNS border regions, T cells need to be reactivated by antigen-presenting cells (APC) to gain access to the CNS parenchyma across the BBB. Production of cytokines/chemokines and subsequent activation of nearby tissue including the blood–meningeal barrier and BBB enables the infiltration of more immune cells and MOG-IgG into the CNS parenchyma that directly damage neurons and glia (reviewed in [Bibr R80][Bibr R81][Bibr R82][Bibr R83][Bibr R84]). Resident DC or infiltrating myeloid cells likely contribute (78,85,86,87,88). Furthermore, CNS border-associated macrophages get highly activated in the course of EAE, which also includes upregulation of major histocompatibility complex (MHC) 2 (89,90).

As different T-cell subsets have diverse roles in immunopathogenesis, it is important to understand toward which lineages T cells are polarized. Different studies examining the cytokine/chemokine profiles in patients with MOGAD measured increased levels of T-helper (Th)17-related cytokines/chemokines (interleukin [IL]-6, IL-8, IL-17a), granulocyte-colony stimulating factor, Th1-related cytokines (interferon-γ, TNFα), and several B-cell associated factors (a-proliferation-induced ligand, B-cell activating factor, C-X-C motif chemokine ligand 13) in cerebrospinal fluid (CSF) and serum (91,92,93).

Tocilizumab (anti-IL-6 receptor antibody) is used off-label for the treatment of AQP4-IgG seropositive NMOSD and because of increased IL-6 levels in the CSF of MOGAD patients (92,93), off-label treatment was evaluated in several case series.

Increased neurofilament light chain levels were observed in the serum of MOGAD patients that also correlated with attack severity and could therefore serve as a potential biomarker (97). In addition, another study found increased CSF myelin basic protein levels in MOGAD and NMOSD patients compared with MS and controls, but glial fibrillary acidic protein levels were only increased in NMOSD (98).

In MOGAD, the detection of MOG-specific T cells is still challenging. One study stimulated patient-derived peripheral blood mononuclear cells of MOG-IgG-positive patients with different MOG peptides, but could not find any specific proliferation. The authors suggest that the use of peptides could be insufficient for T-cell stimulation (99). Another investigation used bead-coupled recombinant MOG for stimulation of T cells in MS patients and observed MOG reactivity in about half of them. However, all patients were treated with natalizumab and only one patient harbored MOG-IgG (100).

As MOGAD is associated with the presence of MOG-IgG, the question arises whether these antibodies are directly pathogenic, or the epiphenomena of a secondary immune response against MOG. Understanding this distinction can help to figure out the role of MOG-specific B cells in disease development. B cells can damage CNS tissue through diverse mechanisms including release of toxic exosomes and cytokines, antigen presentation to T cells, and antibody secretion (reviewed in Refs. 101,^102^). The importance and ability of B cells to sufficiently activate T cells through antigen presentation in EAE mouse studies has revealed contrasting results (103,104). In human MOGAD, one study identified MOG-specific B cells in 60% of patients; still, this did not correlate with MOG-IgG serum titers (105). In contrast, B-cell activation associated with the production of IL-10 in EAE mice has been shown to exert a beneficial effect (106,107). Importantly, IL-10 producing regulatory B cells were reduced in the periphery of MOGAD patients, whereas pro-inflammatory memory B cells, and follicular T cells, that drive B-cell differentiation toward memory cells and long-lived plasma cells, were observed at higher levels (108).

Several T- and B-cell targeting drugs are used off-label in the treatment of MOGAD, including azathioprine and mycophenolate mofetil (109,110,111). One of the most frequently used drugs is rituximab, targeting CD20^+^ B cells (84,109,110,112). However, despite efficient B-cell depletion, only 55% of patients were relapse free in the first and 33% in the second year (113,114). Thus, B-cell depletion was less effective as in AQP4-IgG-positive NMOSD, indicating that B cells may be less important in MOGAD.

## THE ROLE OF HUMAN MYELIN OLIGODENDROCYTE GLYCOPROTEIN-IgG AND NEUROPATHOLOGICAL FINDINGS

As mentioned above, the investigation of the pathogenic potential of human MOG-IgG is hampered by the fact that not all human MOG-IgG cross-react with rodent MOG (26,29,115). Different possible mechanisms for MOG-IgG-derived pathogenicity have been described in the literature. Most MOG-IgG production is believed to take place in the periphery as oligoclonal CSF bands are missing in 90% of MOGAD patients (116). Nonetheless, isolated CSF MOG-IgG positivity was observed in rare cases (117,118). MOG-IgG are primarily IgG1 isotype, but IgG2, IgG3, and IgG4 are sometimes present (119). The role of complement activation in MOGAD is still under debate and not well-established. Only a portion of monoclonal MOG-antibodies was able to activate complement in vivo (120) and injection of human MOG-IgG together with human complement resulted in only low amounts of complement deposition (121). In addition, an ex vivo study found complement activation in only one of 10 samples (29). In contrast, increased serum levels of complement products were found in MOGAD compared with MS, and NMOSD (122). Interestingly, after the transfer of human MOG-IgG cross-reactive to rodent MOG into different rat models, increased T-cell infiltration or complement deposition, together with MOG- or MBP-specific T cells, respectively, was observed (115).

MOG-IgG has shown a direct pathogenic effect on oligodendrocytes: changing the cytoskeleton, repartitioning of MOG into lipid rafts, altering the phosphorylation pattern of different proteins (6,123,124), and furthermore, changing the expression of axonal proteins (121). Moreover, human MOG-IgG induced natural killer-cell-mediated killing of MOG expressing cells in vitro (125) and enhanced antigen presentation through opsonization by APC (126,127).

Systematic neuropathological examinations of patients with MOGAD are rare and include several case reports and 2 larger studies (128,129). The neuropathological examinations of autopsies and biopsies from patients revealed a pattern of perivenous and confluent demyelination present in white matter, the cortex, and in deep gray matter structures (128,129,130). Importantly, confluent lesions were the result of fusion of perivenous lesions rather than MS-like radial expanding lesions. Moreover, slowly expanding plaques, as observed in MS, were missing, and in only one case, a rim of macrophages was present (128). Meningeal inflammation was observed in 86% of a biopsy cohort and furthermore, subpial lesions were present and myelin-laden macrophages/microglial cells were abundantly found within active demyelinating areas (128). In contrast to MS, infiltrating lymphocytes were mainly of the CD4^+^ type with only few B cells and CD8^+^ T cells (128,129,131). Eosinophils and neutrophils were observed in low-to-moderate numbers. Axons were relatively preserved, but reactive astrogliosis was observed without loss of AQP4 staining (128,132). Creutzfeldt-Peter cells were observed in one study (128), but absent in another cohort (129). Complement activation was demonstrated in active lesions, resembling a Pattern II lesion type in some studies (31,128,132,133), yet was largely absent in another investigation of 11 biopsies (129). In addition, destruction of oligodendrocytes was variable, and selective loss of MOG was missing (31,128,132); however, described in another study (129). Premyelinating oligodendrocytes were found in lesions without evidence of active remyelination (31,128). Interestingly, in a study describing the MRI lesion resolution in patients with MOGAD, NMOSD, and MS, MOGAD lesions were found to be resolving completely more frequently compared with the other groups, suggesting better repair capacities (i.e., remyelination and better axonal preservation) (134).

## MYELIN OLIGODENDROCYTE GLYCOPROTEIN-ASSOCIATED DISEASE LESIONS OF THE OPTIC NERVE AND THE VISUAL PATHWAY

**FIG. 2.**
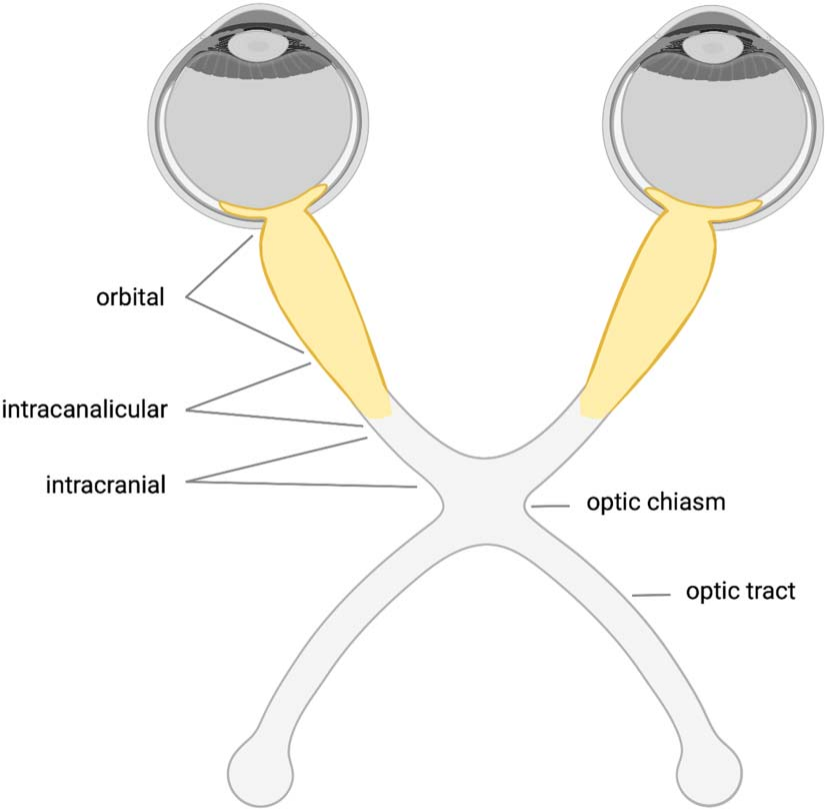
Optic nerve lesions in MOGAD. In MOGAD, bilateral ON is observed in up to 45% of patients and optic disc edema is common. MRI shows enhancement of the optic nerve and perineural abnormalities including optic nerve sheath enhancements in half of the patients. Furthermore, lesions are usually longitudinal extensive and affect predominantly the prechiasmic optic nerve (highlighted in yellow). Involvement of the optic chiasm and the optic tract is only observed in 12% and 2% of patients, respectively. Data from (Refs. 141,^143^). Created with BioRender.com. MOGAD indicates myelin oligodendrocyte glycoprotein-associated disease; ON, optic neuritis

The predominant phenotype in adult MOGAD patients is ON, but it is also frequently found in pediatric cases. Studies reported between 44% and 61% onset presentations with ON in adult MOGAD patients (12,14,16,135) and in up to 38% of children (17,18,19,22). Moreover, studies examining the prevalence of MOG-IgG in ON patients found MOG-IgG in 4%–31% of ON cases (136,137,138,139,140,141). MOG-IgG-positive ON was associated with bilateral ON in 24%–45% of patients (15,16,17,136,137,138,139,142) and pain and optic disc swelling were observed frequently (138,141,143) (Fig. 2). Around half of patients followed a relapsing disease course (15,16,143). Interestingly, of those, around 88% developed isolated ON as relapse, whereas the remaining patients developed NMOSD-like relapses, transverse myelitis, or an optico-spinal phenotype (15,16,143). ON at follow-up was observed in 47% of children and 63% of adult patients with MOGAD (15). Studies reported between 4% and 16% chronic relapsing inflammatory optic neuritis (CRION) patients within MOGAD-ON cohorts and found that CRION patients positive for MOG-IgG were younger, showed bilateral involvement more often and had more relapses compared with seronegative patients (15,143,144). The spectrum of ophthalmic manifestations associated with MOG-IgG is however expanding and therefore, we would like to refer to a recent review (145).

Optical coherence tomography measurements of the peripapillary retinal nerve-fiber-layer (pRNFL) thickness revealed higher values in acute MOGAD-ON compared with MS because of optic disc edema. PRNFL thickening could serve as an indicator to distinguish MOGAD and MS in acute ON (146). After thickening in the acute phase the RNFL and ganglion-cell and inner-plexiform-layer (GCIPL) undergo degeneration (136,147,148), but visual outcome was generally favorable with only 6%–8% showing a poor visual acuity at last follow-up (16,136,143). In MRI of the optic nerve, enhancement was observed in all patients and 50%–88% also showed perineural enhancement. Lesions are usually long, affect the orbital portion more, and can also extend into the orbital fat (141,143,149). Only about 2%–5% developed optic tract abnormalities and 12%–16% showed involvement of the optic chiasm, that was linked to longitudinally extensive lesions in 54% (139,143,150). Prechiasmal and chiasmal lesions were associated with a bad visual prognosis (151).

In EAE animal models immunized with MOG, the lesion distribution was determined by different influences such as gender, the genetic background and the immunization method used (152,153,154). Double transgenic mice (MOG-specific T and B cells, called 2D2/Th) developed spontaneous optico-spinal phenotypes (155,156,157,158). However, single transgenic mice (2D2) also developed ON, although at lower frequencies, suggesting an enhancing role of antibodies (157,159).

Histopathologic examinations of animals revealed infiltration of inflammatory cells, demyelination with axonal loss, and reactive gliosis in retina and optic nerves (152,156,157,160). Besides, complement activation was found in one study (161). The retinal ganglion cell layer was also shown to undergo degeneration in mice after inflammatory responses and activation of microglia cells in later stages of EAE and may be the product of secondary degenerative mechanisms, because there are no MOG-expressing oligodendrocytes present in the retina (157,162,163). As a result, authors observed reduced neuritic density in the inner plexiform layer in mice (157). In contrast, a study examining the pRNFL and GCIPL in MOGAD patients found no evidence for attack-independent degeneration (164). Activation of microglia was furthermore linked to optokinetic tracking threshold decline in functional examinations in experimental autoimmune ON mice (165). In addition, visual evoked potential recordings in dark agouti rats immunized with MOG showed latency delay, a decrease in amplitude, and MOG dose-dependent lack of flash evoked response suggestive of axonal conduction block (160,166). Intriguingly, investigations showed that MOG expression is higher in the optic nerves than in the spinal cord and brain on protein and mRNA levels in mice (155,159). However, the vulnerability of the optic nerve head is likely the result of a lack of microvessels with BBB characteristics and nonspecific permeability in this region (167,168,169).

## CONCLUSION

To summarize, the spectrum of MOGAD-associated symptoms is broad, but most patients present with ON, that is usually associated with a good visual recovery. Histopathology revealed perivenous demyelinating lesions and infiltration of leukocytes. Nevertheless, the role of human MOG-IgG is less clear and different pathogenic mechanisms are discussed. Future studies that aim to define the exact pathogenesis, are needed to further identify targets for efficient treatment strategies.

STATEMENT OF AUTHORSHIP

Conception and design: M. Lerch, M. Reindl; Acquisition of data: Not applicable; Analysis and interpretation of data: Not applicable. Drafting the manuscript: M. Lerch, A. Bauer, M. Reindl; Revising the manuscript for intellectual content: M. Lerch, A. Bauer, M. Reindl. Final approval of the completed manuscript: M. Lerch, A. Bauer, M. Reindl.
